# Temporal Phenolic Profile and Bioactivity of Endemic *Salvia transsylvanica* (Transylvanian Sage) During Flowering

**DOI:** 10.3390/antiox15040417

**Published:** 2026-03-26

**Authors:** Maria-Doroteia Brudiu, Alexandru Nicolescu, Beatriz H. Paschoalinotto, Maria Inês Dias, Gianina Crișan, Andrei Mocan

**Affiliations:** 1 Department of Pharmaceutical Botany, Faculty of Pharmacy, “Iuliu Hațieganu” University of Medicine and Pharmacy, Gheorghe Marinescu Street 23, 400337 Cluj-Napoca, Romania; maria.doroteia.aloman@elearn.umfcluj.ro (M.-D.B.); gcrisan@umfcluj.ro (G.C.); mocan.andrei@umfcluj.ro (A.M.); 2 Laboratory of Chromatography, Institute of Advanced Horticulture Research of Transylvania, Faculty of Horticulture and Business in Rural Development, University of Agricultural Sciences and Veterinary Medicine, Calea Mănăștur 3-5, 400372 Cluj-Napoca, Romania; 3 CIMO, LA SusTEC, Instituto Politécnico de Bragança, Campus de Santa Apolónia, 5300-253 Bragança, Portugal; paschoalinotto@ipb.pt (B.H.P.); maria.ines@ipb.pt (M.I.D.)

**Keywords:** *Salvia transsylvanica*, Transylvanian sage, endemic flora, phenolic accumulation, rosmarinic acid, salvianolic acids, flavonoid glycosides

## Abstract

*Salvia transsylvanica*, an endemic Romanian sage, remains understudied despite co-occurrence with validated medicinal *Salvia* species. In this study, leaves and flowers were harvested weekly during flowering (May and June) and subjected to classical hydroethanolic extraction, HPLC–DAD–ESI/MS^n^ profiling, in vitro antioxidant assays (ABTS, DPPH, FRAP), and enzyme-inhibitory screening, with multivariate analysis correlating compositional patterns with bioactivity. Rosmarinic acid dominated the phenolic profile (68.6 mg/g maximum in week 4), alongside salvianolic acids (salvianolic acid K isomers) and flavonoid glycosides (luteolin, apigenin, and hispidulin hexosides). Leaf extracts displayed higher ABTS/DPPH scavenging (higher phenolic acid content), while flowers showed superior FRAP and α-glucosidase inhibition (IC_50_ 84–143 μg/mL, higher flavonoids), with maximal values being identified during week 4. *S. transsylvanica* offers complementary antioxidant profiles to commercial sages, warranting future in vivo validation for therapeutic applications.

## 1. Introduction

**Figure 1:**
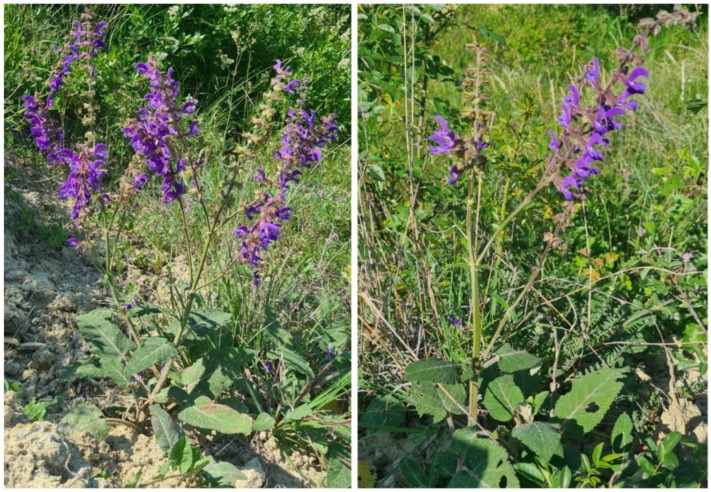
*S. transsylvanica* specimens belonging to a natural population of the endemic flora of Transylvania (Băgău, Alba County, Romania). The species forms part of xerophilous steppe-like grassland present in the region.

*Salvia transsylvanica* (Schur ex Griseb. & Schenk) Schur, commonly denoted as “Transylvanian sage” or “Romanian sage”, is an herbaceous perennial and xeromesophyte species belonging to the Lamiaceae family. It is present in the steppe-like grasslands of the northern and central part of Romania (the Transylvanian Basin and the Carpathian Mountains–the Transylvanian Alps), where it is regarded as part of the endemic flora (Figure 1) [1,2]. The species was recently described as “threatened” based on extinction risk predictions [3] and it is co-distributed and frequently intercrossed with common sage species, including the meadow sage (*S. pratensis* L.) and the woodland sage (*S. nemorosa* L.) [4]. This property was previously used to study the interspecific crossability of *S. transsylvanica* [5]. Within Transylvania, the species can be found along other typical sages, such as the Austrian sage (*S. austriaca* Jacq.) [6].

Despite not being widely recognized as an ethnobotanically relevant species, *S. transsylvanica* shares morphological and ecological characteristics with *S. pratensis*, suggestive of an analogous phytochemical potential [7]. Indeed, although some studies have preliminarily investigated the Transylvanian sage, it remains poorly investigated in terms of its chemical composition and biological properties. Regarding its potential bioactivity, hydroethanolic extracts were shown to possess in vivo analgesic, antipyretic, and anti-inflammatory effects, with low levels of toxicity [8]. Other bioactivity determinations applied to 50% methanol and 70% ethanol extracts suggested in vitro antidiabetic, neuroprotective, antioxidant, antibacterial, and antifungal potentials [9,10].

Previous studies identified some secondary metabolites that are frequently identified in sage species, such as phenolic acids (rosmarinic, caffeic, *p*-coumaric, *p*-hydroxybenzoic, and chlorogenic acid), flavonoids (catechin, epicatechin, rutin, naringin, and quercetin), and terpenoid constituents of essential oil (carvacrol) [9,10]. Despite this fact, there is no available information regarding the comprehensive metabolite composition and quantification of its extracts. Notably, salvianolic acid derivatives, rosmarinyl derivatives, and specific flavone glycosides have not been previously reported for this species, further underscoring the incomplete characterization of its phytochemical profile.

The flowering stage in herbaceous and perennial plants represents a critical developmental stage, associated with significant shifts in secondary metabolite biosynthesis [11]. In Lamiaceae, the phenylpropanoid pathway is responsible for the production of rosmarinic acid, salvianolic acids, and flavonoid glycosides, being modulated by both ontogenic and environmental signals during flowering [12,13]. Studies on related *Salvia* species, such as *S. officinalis*, showed that phenolic accumulation reaches peak levels at specific stages of the flowering period, during the month of May [14,15]. This temporal variability has practical implications for the optimal harvesting of medicinal plant material, as phytochemical composition can differ substantially between early and late flowering stages [13,16]. Despite these observations, the phenolic dynamics of *S. transsylvanica* during flowering have not yet been investigated.

Considering these factors, we hypothesized that (a) the accumulation of phenolic acids and flavonoids in *S. transsylvanica* would vary temporally across the flowering period, leading to organ-specific and time-dependent differences in antioxidant and enzyme-inhibitory bioactivities, and (b) multivariate statistical analysis would reveal mechanistically distinct associations between specific phenolic classes and the measured bioactivities.

To test these hypotheses, this study aimed to: (a) investigate temporal changes in phenolic accumulation during flowering across both leaves and flowers; (b) perform untargeted HPLC-MS^n^ (high-performance liquid chromatography–tandem mass spectrometry) analysis to identify the major phenolic constituents of the extracts; and (c) correlate in vitro antioxidant and enzyme-inhibitory capacity determinations with the phytochemical composition using multivariate statistical analysis. To the best of our knowledge, this is the first comprehensive temporal phytochemical and bioactive report on *S. transsylvanica*, functioning as a foundation for subsequent investigations.

## 2. Materials and Methods

### 2.1. Reagents and Standards

The reagents used for the application of spectrophotometric assays and the analytical standards used for HPLC quantification (≥95% purity) were purchased from Merck KGaA (Darmstadt, Germany).

### 2.2. Plant Material

The leaves and flowers of *S. transsylvanica*, designated as *folium* and *flos*, respectively, were identified by Dr. Mocan Andrei, harvested, and subsequently used for the extraction process. As presented in Table 1, the two categories of plant material were harvested for five consecutive weeks, during the flowering period of the species, specifically on 16, 23, and 30 May and on 6 and 13 June 2018. A representative population was prior identified and selected near Băgău, Lopadea Nouă, Alba County, Romania (geographic coordinates: 46°18′40.4″ N, 23°48′11.6″ E). All plant material was collected exclusively from this single representative ecotype over the mentioned period, which limited the extrapolation of compositional findings to other populations.

Plant material was identified based on botanical data, and the harvesting was applied from endemic flora in accordance with local environmental and ethical regulations, while voucher specimens were deposited in the Herbarium of “Iuliu Hațieganu” University of Medicine and Pharmacy from Cluj-Napoca. Classical drying was applied (temperature 20 ± 2 °C, low-intensity shade, a total of 4–5 days, and moisture content at the end of drying ~10 ± 2%), followed by grinding to a fine powder (sieved using mesh size of 800 µm) using a laboratory mill (6000 rpm, 2.0 min); the plant material was then directly extracted.

### 2.3. Extraction Procedure

The extraction procedure was chosen according to previous studies regarding the ethnobotanical valorization of *Salvia* species from the endemic flora of Romania, examples being the protocols described separately by Mocan et al. and Nicolescu et al. for *S. glutinosa* [9,17]. In conjunction with the ethnobotanical practices of Romania, a traditional hydroethanolic maceration protocol was selected, while also adapting previous studies on Lamiaceae species [18,19]. Dried powder was weighed and mixed with 70% hydroethanolic solution (*v*/*v*), in a 1:10 ratio (*m*/*v*, g per mL), with daily shaking and maceration at 20 ± 2 °C and in the absence of light for a total of ten days. Then, the primary extracts were subjected to vacuum paper filtration and ethanol evaporation using a laboratory rotary evaporator (Heidolph Instruments GmbH & Co. KG, Schwabach, Germany), and the remaining aqueous fraction was freeze-dried (72 h, condenser temperature −55 °C, pressure ≤ 0.1 mbar) using a lyophilizer (Alpha 1–2 LDplus, Martin Christ Gefriertrocknungsanlagen GmbH, Osterode am Harz, Germany), obtaining the final dried extract. The samples were labeled based on the plant material and harvest date, as STA1–5 (for leaf extracts) and STB1–5 (for flower extracts), and stored until further analyses in a desiccator.

### 2.4. Phytochemical Screening

In vitro phytochemical screening was applied using two methods, namely, the total phenolic content (TPC), using the Folin–Ciocalteu method, and the aluminum chloride method for flavonoid quantification, adapted to a microplate reader (SPECTROstar Nano, BMG Labtech, Ortenberg, Germany), as described in previous protocols [17]. The results were expressed as milligrams of gallic acid equivalents per gram of freeze-dried extract (mg GAE/g DW) for TPC and as milligrams of rutin equivalents per gram of freeze-dried extract (mg RE/g DW) for AlCl_3_ method.

### 2.5. Determination of Antioxidant Capacity

In vitro antioxidant potential was determined by using standard methods, namely, for radical scavenging activity (ABTS and DPPH) and for ferric-reducing antioxidant power (FRAP). The protocols were based on previous protocols and adapted to a microplate reader [9,17]. For this, the extracts were solubilized in a 70% hydroethanolic mix to a concentration of 1 mg/mL and then further diluted if necessary. Every result was expressed as mg of Trolox equivalents per gram of freeze-dried extract (mg TE/g DW).

For ABTS, the radical solution of 2,2-azino-bis(3-ethylbenzothiazoline-6-sulfonic acid) was used, prepared by mixing it with potassium persulfate in water solution. After proper dilution, the samples were mixed with the reagent and the absorbances were read after 6 min at λ = 734 nm. For DPPH, the samples were mixed with 2,2-diphenyl-1-picrylhydrazyl solution and the absorbances were read after 30 min at λ = 517 nm. For FRAP, the samples were mixed with the FRAP reagent, a mix of acetate buffer, 2,4,6-tris(2-pyridyl)-*s*-triazine (TPTZ), and ferric chloride, and the absorbances were read after 30 min at λ = 593 nm.

### 2.6. HPLC-MS Analysis of Phenolic Compounds

The separation and identification of phenolic compounds were achieved using a previously described method [17], based on an LC-DAD-ESI/MS^n^ instrumental analysis. For this, the samples were solubilized in a 70% hydroethanolic mix to a stock concentration of 10 mg/mL, ultrasonicated for 10 min at 40 kHz and 25 °C in an ultrasonic bath, filtered through a 0.22 μm PTFE membrane, and further diluted to a working concentration of 1 mg/mL prior to injection into the chromatograph (Dionex Ultimate 3000 UPLC, Thermo Scientific, Waltham, MA, USA). Chromatographic separation was performed on a Waters Spherisorb S3 ODS-2 C18 (3 μm, 4.6 × 150 mm, Waters, Milford, MA, USA) maintained at 35 °C and the injection volume was 10 μL. Detection was achieved at λ = 280, 330, and 370 nm using DAD and mass spectrometry (Trap LTQ XL, Thermo Finnigan, Somerset, NJ, USA). Negative ionization mode (ESI^−^) was selected as it provides optimal sensitivity and selectivity for phenolic acids and flavonoid glycosides (anthocyanins were not a targeted compound class) [20].

The tentative annotation of individual metabolites was based on their chromatographic characteristics and by comparison with available mass spectra (e.g., MassBank) or analytical standards. For data acquisition, the Xcalibur data system (Thermo Fisher Scientific, Waltham, MA, USA) was employed. Quantification was applied using external calibration curves constructed with analytical standards of phenolic compounds, with expression as mg compound/g DW of freeze-dried extract. Specifically, the following standards were used for calibration: caffeic acid, gallic acid, rosmarinic acid (for phenolic acids), quercetin-3-*O*-glucoside, and apigenin-7-*O*-glucoside (for flavonoid-hexosides). Quantification was performed in duplicate (*n* = 2) due to sampling constraints; while this limited statistical power for individual compound comparisons, the duplicates served to verify chromatographic reproducibility.

### 2.7. In Vitro Enzyme Inhibition Assays

In vitro enzymatic inhibition assays were applied, as previously described [17,21]. For all assays, the freeze-dried extracts (prepared as described in Section 2.3) were re-solubilized in a 10% DMSO water solution, filtered, and then further diluted.

The α-glucosidase inhibition assay was applied for the assessment of antidiabetic potential, based on the formation of *p*-nitrophenol from *p*-nitrophenyl-α-D-glucopyranoside (PNPG). In short, 50 μL of re-solubilized extract (in the concentration range of 270–30 μg/mL for flower extracts and 645–80 μg/mL for leaf extracts) was mixed with 50 μL of enzyme solution (0.75 U/mL in phosphate buffer with pH = 6.8), 50 μL of phosphate buffer, and 50 μL of the substrate (PNPG, 10 mM in the same phosphate buffer). Absorbance was measured at λ = 405 nm after 5 min of incubation at 37 °C.

The acetylcholinesterase inhibition assay was based on Ellman’s method. In brief, 25 μL of re-solubilized extract (in the concentration range of 1600–130 μg/mL for flower extracts and 1700–140 μg/mL for leaf extracts) was mixed with 50 μL of 50 mM Tris-HCl buffer (with pH = 8.0), 125 μL of 0.9 mM 5,5′-dithiobis-2-nitrobenzoic acid (DTNB) solution (in Tris-HCl buffer), and 25 μL of enzyme aqueous solution (0.078 U/mL). After 15 min of incubation at 25 °C, 25 μL of 4.5 mM acetylthiocholine iodide solution (in Tris-HCl buffer) was added and then the reaction mix was re-incubated for another 10 min. Absorbance was measured at λ = 405 nm.

The tyrosinase inhibition assay, an initial screening of samples at high concentrations (10 mg/mL), did not provide any inhibition effects, and the method was not further applied.

The percentage of inhibition or I% was determined based on the following equation:

$$I\%=100\times \left(\frac{Ac-As}{Ac}\right),$$

where Ac and As represent the absorbances of the control (complete enzymatic reaction, with solvent blank subtracted) and the sample (inhibited enzymatic reaction, with sample blank subtracted), respectively. The results were expressed as IC_50_ values, based on normalized logarithmic curves of determined I%, with acarbose and galantamine serving as therapeutic standards.

### 2.8. Statistical Analysis and Software

Each in vitro determination was performed in triplicate and expressed as a mean value ± standard deviation (*n* = 3). For the assessment of significant statistical difference between samples, one-way ANOVA was used with post-hoc analysis (Tukey’s test); statistical significance was considered at *p* < 0.05. For LC–MS quantification, values (*n* = 2) for each given metabolite (row) were compared between extracts (columns) using two-way ANOVA with simple-effects post-hoc analysis (Tukey’s test). Logarithmic plotting of logC values against I% for enzyme inhibition determinations was performed using GraphPad Prism version 8.0.

Heatmap with hierarchical clustering (Euclidean distance, complete linkage, applied to both rows and columns) and Pearson correlation coefficients (*r*, reported in Section 3.6) were performed using RStudio software (version 2026.01.1) within the ‘pheatmap’ package [22]. PCA was performed using the ‘prcomp’ function applied to z-score standardized variables, and the biplot was constructed using the ‘ggplot2′ [23] and ‘ggrepel’ packages, representing sample scores and variable loadings for the first two principal components.

## 3. Results and Discussion

### 3.1. Phytochemical Screening

In this study, rapid colorimetric screening was applied to assess the presence of distinct classes of phenolic compounds, namely, total phenolic content (TPC) and flavonoid content (TFC).

**Figure 2:**
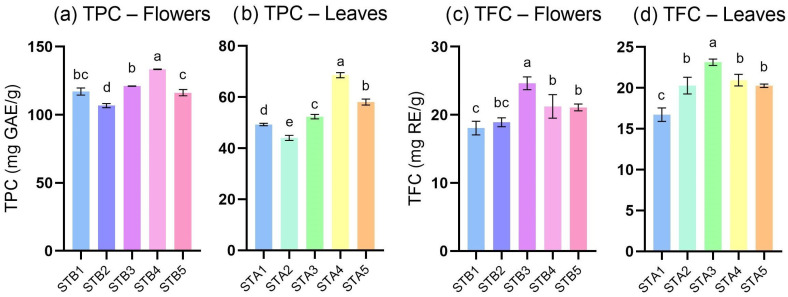
Histogram representation of values obtained for phytochemical screening, using TPC—total phenolic content (**a**,**b**) and TFC—total flavonoid content (**c**,**d**). Statistical variance in each case was assessed using one-way ANOVA with post-hoc analysis (Tukey’s method), where different letters represent statistically significant differences (*p* < 0.05).

Regarding TPC (Figure 2a,b), the results were completely discriminated according to the plant organ, with the flower extracts showing significantly higher values (106.68 ± 1.52–133.40 ± 0.15 mg GAE/g DW) in comparison to the leaf extracts (44.03 ± 0.98–68.57 ± 0.97 mg GAE/g DW). Moreover, the comparison of STA and STB pairs showed *p*-values < 0.01 for TPC, suggesting that the overall phenolic species were concentrated in the flowers. The highest values were identified for the STA4 and STB4 samples, indicating a possible maximal accumulation of phenolic metabolites during the fourth week of flowering. These temporal maxima were significantly higher than the corresponding samples STA3/5 and STB3/5 (*p*-values < 0.0001). The obtained values are in line with previous studies; for example, Luca et al. recently determined the TPC of several extracts of Eastern European sage species, with values ranging from 57.87 to 126.91 mg GAE/g DW (for *S. austriaca* Jacq. and *S. officinalis* L., respectively) [24]. At the same time, hydroethanolic extracts of *S. transsylvanica* aerial parts showed TPC values of 20.32 mg GAE/g DW [9], while Romanian *S. glutinosa* L. hydroethanolic macerates presented values of 169.14 and 178.65 mg GAE/g DW [17].

On the other hand, the TFC values using the AlCl_3_ assay could not differentiate between the two types of plant organs, collected at the same period, with *p* > 0.05 in all cases. The values ranged between 16.72 ± 0.83 mg RE/g DW (STA1) and 24.62 ± 0.94 mg RE/g DW (STB3), with the highest values being identified for the STA3 and STB3 samples, representing the third week of flowering. As in the case of TPC, this pair of samples were significantly higher in TFC than the other samples, at *p*-values < 0.02. The obtained values are in line with previous determinations applied to hydroethanolic extracts of *S. glutinosa* (22.75 and 30.16 mg RE/g DW) [17] and *S. transsylvanica* (13.94 mg RE/g DW) [9].

No statistically significant correlation was observed between TPC and TFC values (*r* = 0.20), suggesting divergent biosynthesis during the flowering, with distinct patterns of accumulation between the leaves and flowers. Moreover, the mechanism of the AlCl_3_ method partly explains the high affinity of AlCl_3_ for flavones and flavonols with clear substitution patterns and should not be regarded as a comprehensive quantification of flavonoids [25]. The results of TPC and TFC confirmed the presence of phenolic species in high quantities, which oriented the study in the direction of their separation and identification using HPLC-MS.

### 3.2. HPLC-MS Separation and Identification of Phenolics

Separation and identification of phenolic compounds (Table 2) was accomplished using HPLC–DAD–ESI/MS^n^, allowing for the putative annotation of two major classes of metabolites: phenolic acids and flavonoids. As expected, these secondary metabolites are typical for sage species, with some of them being frequently encountered in the taxa of Lamiaceae family [26].

#### 3.2.1. Phenolic Acids and Derivatives

Phenolic acids were annotated as monomers and esters of hydroxycinnamic acids (rosmarinic acid and its derivatives), including trimer phenolics. First, salvianic acid A or danshensu (**1**) was tentatively annotated based on the fragmentation from [M−H]^−^ at *m*/*z* 197 to *m*/*z* 179, and to 135, respectively [27,28]. Compound **2**, present in all samples, was annotated as a gallic acid (galloyl) derivative, based on the common fragment at *m*/*z* 169, yet it remained unidentified, similarly to a previous study [29]. Caffeic acid (**4**) was identified based on the [M-H]^−^ ion at *m*/*z* 179 and previously injected standards [30]. Interestingly, two less frequent phenolic acids were annotated. Caffeoyl-threonic acid (**3**), structurally an ester derivative of caffeic acid, was identified based on the ion [M-H]^−^ at *m*/*z* 297 and the fragment ions at *m*/*z* 179, 161, and 135 [31]. An isomer of yunnaneic acid type E (**5**) was identified based on fragmentations from *m*/*z* 571 to 553, 527, and 179 [32,33,34].

Rosmarinic acid, a caffeic acid ester, was identified along with three of its derivatives, based on similar fragmentation patterns. The acid itself (**12**) presented a pseudo-molecular ion [M-H]^−^ at *m*/*z* 359, followed by specific fragments in MS^n^ at *m*/*z* 197, 179, 161, 135, and 123 [35,36]. Two isomers of rosmarinic acid or rosmarinyl hexosides (**8** and **9**) were annotated based on fragmentation from *m*/*z* 521 for [M-H]^−^ ion to the specific fragments of rosmarinic acid (*m*/*z* 359, 197, 179, and 161), with an initial loss of 162 u.

Similarly, compound **19** was annotated as methyl rosmarinate, based on the initial [M-H]^−^ ion at *m*/*z* 373, followed by fragmentation to *m*/*z* 197, 179, 161, and 135 [27].

Salvianolic acids, dimers and trimers of phenolic acids, were identified in Transylvanian sage extracts as three possible isomers of salvianolic acid K (compounds **6**, **7**, and **11**), representing ester derivatives of danshensu (salvianic acid A) with caffeic acid. These isomers were annotated based on the common [M-H]^−^ ion at *m*/*z* 555, followed by different fragments, such as 537, 493, 395, 357/358, 313, 295, 197, and 179, according to available literature data [31,35,37,38]. Lastly, one isomer of salvianolic acid I or melitric acid A (**20**) was tentatively annotated, based on its specific fragmentation patterns from [M-H]^−^ ion at *m*/*z* 537 to 493, 359, 197, 179, 161, and 135 [37,39,40].

#### 3.2.2. Flavonoid Compounds

Flavonoid compounds were exclusively annotated as flavonoid glycosides derived from flavones (apigenin, luteolin), methoxylated flavones (an isomer of hispidulin), and flavonols (kaempferol), according to previous studies [31,37,41]. First, luteolin-*O*-hexoside (**10**) was identified based on the loss of 162 u from the pseudo-molecular ion [M-H]^−^ at *m*/*z* 447 to 285 (representing luteolin aglycone), followed by specific fragments at *m*/*z* 199, 175, and 151. The acetylated derivative of this flavonoid, luteolin-*O*-acetyl-hexoside, was tentatively annotated in the form of two isomers (**16** and **17**), based on similar fragmentations, presenting the [M-H]^−^ ion at *m*/*z* 489 (a loss of 42 u to *m*/*z* 447 and further fragmentation to *m*/*z* 285) [17].

Similarly, kaempferol-*O*-acetyl-hexoside (**14**) was identified based on its aglycone with *m*/*z* 285 and fragmentation at *m*/*z* 175, while the fragmentation of the glycosidic part was denoted by ions at *m*/*z* 489 and 447. Compounds **13** and **18** were annotated as apigenin derivatives, namely, apigenin-*O*-hexoside and apigenin-*O*-acetyl-hexoside, based on the common [M-H]^−^ ion at *m*/*z* 269 of the flavone aglycone. The losses specific to the glycosidic parts were represented by transitions from *m*/*z* 473 to 431 and 269, respectively, followed by a fragment of apigenin identified at *m*/*z* 227. An interesting example was compound **15**, which presented an ion at *m*/*z* 299 and further ions at *m*/*z* 284 and 227, suggesting it is a mono-methoxylated flavone or flavonol derivative. Moreover, the transition from *m*/*z* 461 to 299 indicates the presence of a hexoside moiety. Considering its occurrence in sage species, it could be suggested that either hispidulin, or another methoxy-apigenin isomer, represent this compound [42,43].

### 3.3. HPLC-MS Quantification of Phenolics

The individually separated phenolic metabolites were also quantified based on available standards using an adapted HPLC-DAD method (Table 3).

#### 3.3.1. Total Phenolic Compounds

As observed, in terms of the overall temporal accumulation of phenolic metabolites, the leaf extracts showed the highest total quantities of phenolic species (139.4, 126.4, 114.4, 96.8, and 81.3 mg phenolics/g extract, *p* < 0.0001 between all), decreasing in the order STA4 > A5 > A3 > A1 > A2. At the same time, relatively lower values were identified for the flower extracts (103.0, 97.2, 81.4, 79.8, and 71.3 mg phenolics/g extract, *p* < 0.0001 between all), decreasing in the order STB4 > B2 > B5 > B3 > B1. Nonetheless, the range of 71.30–139.41 mg phenolics/g extract corresponds to an almost double maximal accumulation in leaves in comparison to flowers. Similarly to the results of the TPC and TFC determinations, these cumulative values indicate that the anabolic activity of the plant yielded the highest quantities of phenolic metabolites during the fourth and fifth week of flowering, specifically between 6 and 13 June.

At the same time, it is important to note that total phenolic quantification (higher in leaves) and the TPC values (higher in flowers; see also Section 3.1) appear contradictory. This discrepancy can be attributed to the non-selective nature of the TPC reagent, which reacts with all reducing compounds present in the extract (e.g., reducing sugars and phenolic polymers such as tannins) [44,45]. Presumably, flower extracts may contain a higher content of such reducing species alongside the quantified phenolics, leading to higher TPC values compared to HPLC-based quantification.

#### 3.3.2. Total Phenolic Acids

Regarding total phenolic acids, with values between 66.0–127.0 mg/g extract, the highest quantities were observed for the samples STA4, STA5, and STA3 (significantly different with *p* < 0.0001), representing leaf extracts for plant material harvested at the end of May and early June. In this class, the most abundant metabolite was rosmarinic acid (**12**, with the highest value of 68.6 mg/g in STA4), followed by an isomer of salvianolic acid K (**11**, maximal 26.50 mg/g in STA5), methyl rosmarinate (**19**, maximal 16.4 mg/g in STA3), salvianolic acid I (**20**, maximal 6.9 mg/g in STA4), and rosmarinyl hexoside (**8**, maximal 5.5 mg/g in STA4). While all these metabolites were highly concentrated in the leaves, they were also present in the flower extracts. Interestingly, another isomer of salvianolic acid K (**6**) was only identified in the flowers.

**Figure 3:**
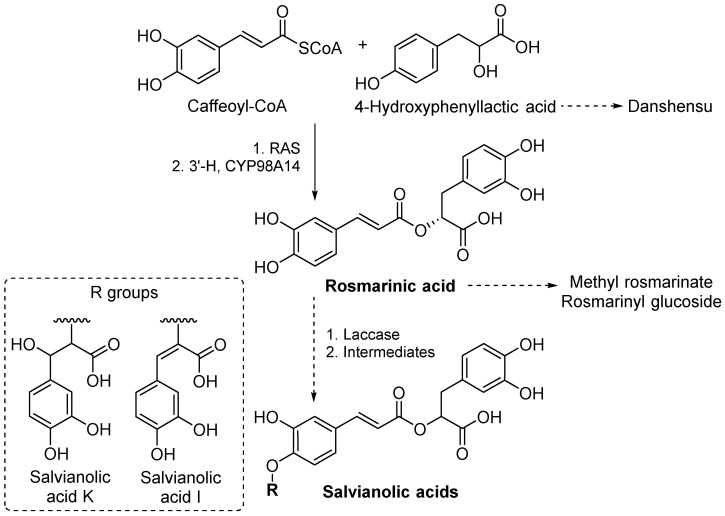
Metabolic pathways including the highly abundant phenolic acid metabolites identified in *S. transsylvanica* extracts of leaves and flowers. Intermediates in salvianolic acid synthesis are represented by phenoxyl radicals and quinones. 3′-H: hydroxycinnamoyl-hydroxyphenyllactate 3′-hydroxylase; RAS: rosmarinic acid synthase [47,49,50,51].

Chemically, the highly abundant phenolic acids were exclusively represented by dimer (as rosmarinate derivatives) and some trimer derivatives of caffeic acid, known as salvianolic acids [46]. These metabolites are biosynthesized through a common biochemical anabolism, starting in parallel from the phenylpropanoid and tyrosine-derived pathways, with rosmarinic acid serving as a central metabolite (Figure 3). In the first step, caffeoyl-CoA and 4-hydroxyphenyllactic acid (4-HPLA) are condensed, in the presence of rosmarinic acid synthase (RAS), to the intermediate caffeoyl-4′-HPLA, which is subsequently 3′-hydroxylated to rosmarinic acid under the action of hydroxycinnamoyl-hydroxyphenyllactate 3′-hydroxylase, belonging to the class of CYP98A14 hydroxylases [47,48]. The conversion of rosmarinate to salvianolic acids takes place through the action of laccase and subsequent rearrangements of phenoxyl radicals and quinones [49,50,51]. It can be hypothesized that the abundance of phenolic acids during the fourth week of harvesting could be linked to an upregulation of phenylpropanoid pathways, possibly characteristic during flowering [52].

Rosmarinyl hexosides, with isomers such as salviaflaside (rosmarinic acid 3′-*O*-glucoside) being specific metabolites for Lamiaceae, are obtained through glycosylation reactions [13]. On the other hand, biosynthesis of methyl rosmarinate is poorly characterized and could take place through enzymatic esterification.

#### 3.3.3. Total Flavonoid Species

Regarding total flavonoids, ranging between 4.50–16.60 mg/g extract, the highest quantities were observed for the samples STA3, STB2, STA5, and STB5. In the case of leaf extracts, the end of May and early June period was the most relevant for higher content, while for the flower extracts no apparent pattern was identified.

Individual quantitative differences were also observed between flowers and leaves. Luteolin-*O*-hexoside (**10**) was the most abundant flavonoid, with values between 2.3 and 6.3 mg/g extract and the highest concentration in STA3, STA4, STA5, STB3, STB5, and STB2. At the same time, an isomer of hispidulin-*O*-hexoside (**15**) was higher in leaves (with 5.0 mg/g for STA3), while apigenin-*O*-hexoside (**13**) was much higher in flowers (with 7.7 mg/g for STB5). Belonging to the class of flavone derivatives, these metabolites were previously identified in other *Salvia*, such as the frequent apigenin and luteolin glucosides in *S. officinalis* [53,54], as well as hispidulin in *S. fruticosa* [55] and hispidulin glucoside in *S. plebeia* [43].

#### 3.3.4. Qualitative and Quantitative Differences

The literature data regarding the phenolic composition of *S. transsylvanica* are scarce. As such, qualitative and quantitative results complete the gaps regarding its phytochemistry, in conjunction with the observations of Janicsák et al. and Mocan et al., who identified both phenolic acids (caffeic, chlorogenic, *p*-hydroxybenzoic, *p*-coumaric, and rosmarinic acid) and some flavonoid species (catechin, epicatechin, rutin, naringin, and quercetin) [9,10].

In comparison to other closely related species, *S. transsylvanica* showed a similar phytochemical profile. Taking *S. pratensis* as an illustrative example, 31.2 mg of rosmarinic acid/g dry mass was obtained in a Soxhlet 70% alcohol extract [56], while the macerate of aerial parts contained caffeoyl hexosides, danshensu hexoside, caffeoyl-threonic acid, salvianolic acids (A, C, E, and F), rosmarinic acid, methyl rosmarinate, as well as apigenin, luteolin, and kaempferol derivatives [57]. At the same time, *S. nemorosa* extracts contain the same caffeoyl-threonic acid, caffeic acid, rosmarinic acid, methyl rosmarinate, and salvianolic acid K, with hispidulin, luteolin and apigenin mono- and diglucosides being abundant [24,58,59].

### 3.4. Antioxidant Activity

The assessment of antioxidant activity was applied using conventional in vitro assays, based on two different mechanisms: radical scavenging potential (ABTS, DPPH) and metal reducing antioxidant potential (FRAP).

Regarding radical scavenging potential (Table 4), the results indicated that leaf extracts presented the highest activity, with maximal values identified in both assays for the ST A4 sample (139.44 ± 0.58 mg TE/g for ABTS and 92.24 ± 0.33 mg TE/g for DPPH). Nonetheless, the other extracts also showed relevant antioxidant potential, with ST B4 presenting the highest values among flower extracts (90.36 ± 1.15 mg TE/g for ABTS and 77.94 ± 0.39 mg TE/g for DPPH).

On the other hand, for FRAP, the flower extracts showed slightly higher values in comparison to the leaf extracts, being in the range of 236.67 (ST A2)—319.88 (ST B3) mg TE/g DW. Statistical analysis revealed that most of the flower extracts were not statistically different from each other, with the only noticeable exception being ST B3 versus ST B1 (*p* < 0.0001).

Information regarding the antioxidant potential of *S. transsylvanica* is scarce. For 50% aqueous-methanol extracts, Janicsák et al. determined similar EC_50_ values (6.70 μg/mL) with the extracts of *S. officinalis* f. *albiflora* (7.04 μg/mL), exploring the inhibition of auto-oxidation of unsaturated fatty acids in a rat-brain homogenate. Consequently, the extracts of *S. dumetorum* and *S. scabiosifolia* showed similarly important activities [10]. At the same time, the DPPH scavenging activity as IC_50_ of 70% hydroethanolic *S. pratensis* extract was 23.8 µg/mL dry mass [56]. Comparably to our results, Mocan et al. determined antioxidant potentials of 59.29 ± 2.37 (DPPH), 77.53 ± 2.25 (ABTS), and 90.94 ± 2.55 (FRAP) mg TE/g DW, for the 70% hydroethanolic extracts of *S. transsylvanica* from Romania [9].

As previously suggested by Mervić et al., who comparatively studied the bioactivity of Mediterranean sage species, the antioxidant and enzyme-inhibitory activities of *Salvia* extracts can be explained by multiple mechanisms, with the bioactive phenolic profile being of major importance [60]. In particular, the highly abundant rosmarinic acid might contribute to the antioxidant capacity of *S. transsylvanica* due to its chemical structure and bioactive mechanisms [61]. The presence of two catechol or *ortho*-dihydroxy phenyl moieties (originating from the caffeic acid and 3,4-dihydroxyphenyllactic acid monomers) induces favorable effects on the reducing power of the molecule [62,63].

### 3.5. Enzyme Inhibition Activities

Enzyme inhibition potential was assessed against α-glucosidase and acetylcholinesterase using in vitro assays (Table 5). The tested extracts did not exhibit any activity against tyrosinase.

**Figure 4:**
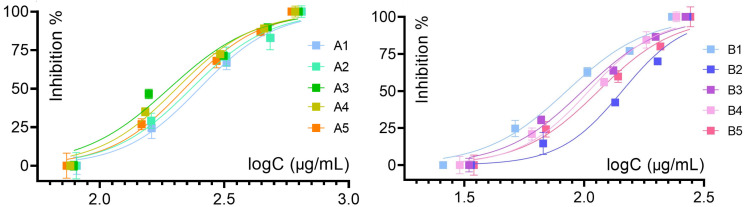
Logarithmic inhibition curves for in vitro alpha-glucosidase inhibition assay, determined for STA—leaf extracts, and STB—flowers extracts. Data presented as mean ± standard deviation (*n* = 3).

The IC_50_ or half-maximal inhibitory concentrations were derived from sigmoidal inhibition curves (illustrated in the case of α-glucosidase in Figure 4), where flower extracts displayed an approximative 2-fold rise in potency over leaf extracts (half-maximal logC ~2.0 vs. logC ~2.4 μg/mL).

Important inhibitory potential was identified for the α-glucosidase assay, where the flower extracts displayed the lowest IC_50_ values, with the activities decreasing in the order STB1 > B3 > B4 > B5 > B2. The lowest IC_50_ value of 84.48 ± 2.89 μg/mL corresponds to the flowers at the beginning of the flowering period (STB1). At the same time, the leaf extracts displayed a diminished activity, decreasing in the order STA3 > A4 > A5 > A2 > A1. These results are comparable to the therapeutic standard acarbose, with an IC_50_ value of 122.27 μg/mL, suggesting potential antidiabetic applications for *S. transsylvanica*, in a similar manner to *S. officinalis* [9,64].

On the other hand, the potential inhibitory effect against acetylcholinesterase was not relevant in comparison to the galantamine standard. The most active sample was STB4, with an IC_50_ value of 535.5 ± 27.39 μg/mL, followed by STB2 and B1.

The anti-glucosidase potential has been previously assessed by Mocan et al., who found that the activity of *S. transsylvanica* hydroethanolic extracts (25.62 ± 1.10 mmol ACAE/g dw) is comparable to that obtained from *S. officinalis* (27.01 ± 0.12 mmol ACAE/g dw) [9]. Furthermore, various sage species from the Balkan region vegetation were shown to possess relevant inhibitory activity against α-glucosidase, with illustrative examples being *S. glutinosa* [17,60], *S. multicaulis*, *S. santolinifolia*, *S. dracocephaloides*, and *S. eremophila* [65]. The activity might be attributed to highly active phenolic components, such as flavonoids (luteolin, hispidulin, and apigenin derivatives) and some phenolic acids (rosmarinic, caffeic, chlorogenic acid) [17,60,65]. Furthermore, previous research identified significant antidiabetic effects related to the administration of rosmarinic acid, such as through hyperglycemia reduction and insulin sensitivity amelioration in diabetic rats [66] and the attenuation of palmitate-induced insulin resistance in muscle cells [67].

### 3.6. Multivariate Statistical Analysis

**Figure 5:**
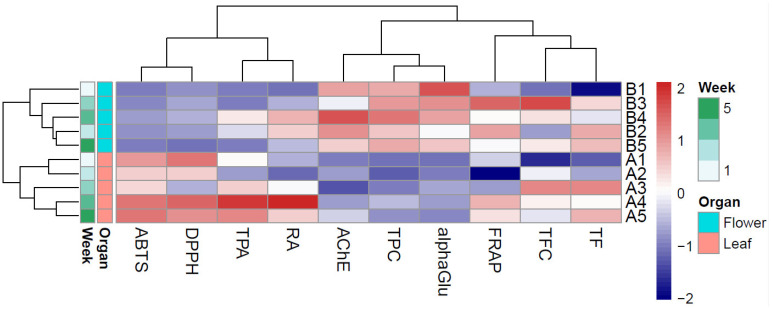
Heatmap with hierarchical cluster analysis of the assessed parameters and each extract. Columns indicate in vitro bioactive potential and phytochemical profiling, while rows indicate the type of the extract, further sorted by organ type (leaf: A or flower: B) and harvesting period (week 1–5). Notations: AChE (acetylcholinesterase inhibition, expressed as 1/IC_50_), alphaGlu (alpha-glucosidase inhibition, expressed as 1/IC_50_), TF (total flavonoids, HPLC), TPA (total phenolic acids, HPLC), TPC (total phenolic content, Folin-Ciocâlteu), TFC (total flavones and flavonols, AlCl_3_), and RA (rosmarinic acid).

A heatmap with hierarchical clustering (Figure 5) was generated to comprehensively assess the relationship between the phenolic profile and in vitro bioactivities of the extracts. As observed from the dendrogram, flower extracts (STB1–5) were clearly separated from leaf extracts (STA1–5), with the flower cluster presenting higher standardized values for total phenolic content (TPC), total flavonoid content (TFC), FRAP, and anti-enzymatic potential (AChE and alphaGlu were transformed as 1/IC_50_ to reflect potency, since lower IC_50_ values indicate stronger inhibition and statistical analysis was aimed for direct correlations). Within this cluster, extracts representing weeks 3 and 4 showed the most potent overall profile. On the other hand, leaf extracts showed more heterogeneous clustering, with samples STA3–STA5 outperforming samples STA1 and 2.

Furthermore, the leaf cluster presented higher standardized values for radical scavenging activities (both ABTS and DPPH) and for the content in phenolic acids (total phenolic acids–TPA, as well as individual rosmarinic acid content–RA). These variables formed a tight cluster, indicating strong co-variation across samples (*r* = 0.85 between ABTS and DPPH, and *r* = 0.97 between TPA and RA). Within this group, the samples STA4 and 5 outperformed the other leaf extracts. Interestingly, for leaf extracts, the values of TFC and cumulative total flavonoids (TF) were highly correlated (*r* = 0.85), highlighting the affinity of AlCl_3_ method in quantifying flavones and flavonols.

**Figure 6:**
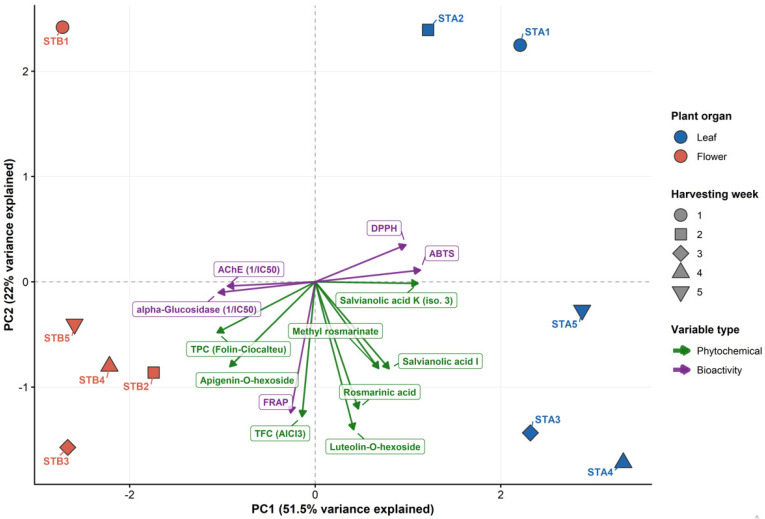
PCA biplot illustrating organ-specific phenolic and bioactivity profiles in *S. transsylvanica* leaf (STA1–STA5; blue) and flower extracts (STB1–STB5; red). Arrows represent z-score-standardized data loadings (green arrows = phytochemical variables, purple arrows = bioactivity variables). Variables: AChE (acetylcholinesterase inhibition, expressed as 1/IC_50_), alpha-Glucosidase (alpha-glucosidase inhibition, expressed as 1/IC_50_), TPC (total phenolic content, Folin-Ciocâlteu), TFC (total flavones and flavonols, AlCl_3_), rosmarinic acid, salvianolic acid K (isomer 3), methyl rosmarinate, salvianolic acid I, luteolin-*O*-hexoside, apigenin-*O*-hexoside.

The PCA biplot (Figure 6) confirmed and extended these organ-specific patterns through unsupervised dimensionality reduction, with the first two PCs explaining 73.5% of the total variance. PC1 (51.5% of total variance) clearly separated leaf extracts (positive scores) from flower extracts (negative scores), representing the primary chemotype axis among different organs. The loading arrows for rosmarinic acid, salvianolic acid K (isomer 3), methyl rosmarinate, and salvianolic acid I pointed in the same direction as ABTS and DPPH, with higher values in the case of leaf extracts; thus, a direct association was established between these individual phenolic acids and radical scavenging capacity, as in the case of the heatmap analysis.

In contrast, along the opposite side of the PC1 axis, the loading vectors for TFC, TPC, FRAP, apigenin-*O*-hexoside, and α-glucosidase inhibition were co-directed toward the scores of flower extracts. This association indicates that flavonoid-enriched flower extracts, characterized by higher apigenin-*O*-hexoside and TFC values, drive FRAP and enzyme-inhibitory potential.

PC2 (22.0% of total variance) resolved intra-organ temporal variation, with the weeks 3 and 4 clustering toward higher bioactivity loadings in both organ groups, which translates into the peak phenolic accumulation identified during the fourth harvesting week. Moreover, luteolin-*O*-hexoside, the most abundant flavonoid, presented higher PC2 loadings toward the third week extracts, being relatively higher in the leaf extracts.

Collectively, these multivariate patterns demonstrate that the bioactive potential of *S. transsylvanica* extracts is governed by two mechanistically distinct and organ-specific chemotype axes. First, phenolic acids (dominated by rosmarinic acid, methyl rosmarinate, and salvianolic acids), identified as the major metabolites in leaf extracts, presented selective radical scavenging effects; this observation is consistent with hydrogen atom transfer (HAT) or single-electron transfer (SET) mechanisms, favorable for hydroxyphenolic acids [62,68]. Second, flavonoids (characterized by flavone glycosides), enriched in flowers, showed increased FRAP and α-glucosidase inhibitory potential, which highlighted their electron donation capacity in the FRAP assay [25,69,70]. These observations can guide targeted isolation and structure–activity studies on *S. transsylvanica* metabolites, especially rosmarinic acid and luteolin-*O*-hexoside.

### 3.7. Transylvanian Sage as a Source of Antioxidant Metabolites

Romania’s flora is represented by an important fraction of the *Salvia* genus in Europe (counting around 36 species) [71], with approximately 15 species being distributed across the country’s diverse ecological areas. Their diversity reflects adaptation to distinct microhabitats within the country’s temperate climate, from open grasslands (*S. austriaca*, *S. nemorosa*, *S. nutans*, *S. pratensis*, *S. sclarea*, *S. verbenaca*, *S. verticillata*), coastal-dry environments (*S. aethiopis*, *S. amplexicaulis*, *S. ringens*), to shady forest margins (*S. glutinosa*) and steppe-like communities, representing both native and introduced–cultivated species (*S. farinacea*, *S. officinalis*, *S. splendens*) [1,72].

The “Transylvanian sage” or “Romanian sage” can be regarded as a distinctive native species in Romanian flora. The results of bioactive and phytochemical analysis suggested the therapeutical potential of aerial parts hydroethanolic extracts, which can be further explored through different approaches (e.g., the optimized extraction of phenolic components, structure–activity relationship analyses, and comprehensive and/or untargeted phytochemical profiling).

Multivariate analysis highlighted correlations between major metabolites (rosmarinate derivatives, salvianolic acids, and flavone-hexosides) and in vitro antioxidant and anti-glucosidase potential of the extracts, while suggesting organ-specific chemotype differences. Rosmarinic acid and methyl rosmarinate, the dominant phenolic fraction of all extracts, are dimers deriving from caffeic acid and 3,4-dihydroxyphenyllactic acid; they present potent antioxidant capacity due to the presence of two catechol (*ortho*-dihydroxyphenyl) moieties, providing four phenolic hydroxyl groups capable of donating hydrogen atoms to free radicals and stabilizing the resulting phenoxyl radical through resonance delocalization [62,68]. This structural feature places rosmarinic acid among the most active natural hydroxycinnamic acid antioxidants [73,74,75,76].

Furthermore, salvianolic acids K and I are oligomeric rosmarinic acid derivatives and thus contain more catechol units, contributing to their radical scavenging and antioxidant capacity [77]. In the case of major flavonoids, namely luteolin- and apigenin-*O*-hexosides, the antioxidant potential is not only conferred by the presence of the catechol B-ring, but also based on chelation mechanisms [69,78].

Beyond the discussed regional context, the phenolic profile of *S. transsylvanica* shares notable similarities with globally recognized *Salvia* species. Rosmarinic acid dominance, alongside salvianolic acids and flavone glycosides, is typical for *S. rosmarinus* (formerly *Rosmarinus officinalis*) [79] and *S. verticillata* [34], while *S. miltiorrhiza* is a rich source of salvianolic acids, particularly A and B isomers [80]. This composition provides a broader chemotaxonomic relevance to *S. transsylvanica*, as oligomeric rosmarinate derivatives have been predominantly reported in sage species used in traditional medicine.

This study also presented methodological limitations, including: (a) the use of plant material collected from a single natural population and a single flowering season (2018), which precludes the assessment of inter-population variability or year-to-year climatic effects on phytochemical composition; (b) an exclusive reliance on in vitro antioxidant and enzyme inhibition assays solely based on hydroethanolic extracts; and (c) untargeted LC–MS, which only allowed for tentative identification of several metabolites. Future research should also consider in vivo, pharmacokinetic, and toxicity studies to validate the translational relevance of the observed bioactivities.

## 4. Conclusions

This study characterized *Salvia transsylvanica*, the Transylvanian sage, using untargeted HPLC-ESI-MS^n^ phenolic profiling, antioxidant capacity assays, and enzyme-inhibitory methods. The 70% hydroethanolic extracts demonstrated high contents in phenolic acids (rosmarinic acid, methyl rosmarinate, three isomers of salvianolic acid K, and salvianolic acid I), alongside flavonoid glycosides (luteolin, apigenin, and hispidulin hexosides), some of which were identified for the first time in this species. Peak phenolic accumulation was observed during late May and early June (weeks 3–4 of flowering), with phenolic acids predominantly concentrated in leaves and apigenin-*O*-hexoside enriched in flowers.

The extracts indicated high antioxidant capacities, assessed through ABTS, DPPH, and FRAP assays, while flower extracts demonstrated potent α-glucosidase inhibitory activity comparable to acarbose. Multivariate analyses revealed two mechanistically distinct chemotype axes: phenolic acids (rosmarinic acid, salvianolic acids) driving radical scavenging in leaf extracts, and flavone glycosides (luteolin- and apigenin-*O*-hexosides) showing ferric-reducing capacity and α-glucosidase inhibition in flower extracts. These findings were based on a single wild population and in vitro evidence, and future in vivo, pharmacokinetic, and multi-population studies are needed to validate their translational relevance. *S. transsylvanica* represents a complementary source of bioactive phenolic compounds to widely recognized sage species, warranting further extraction optimization and bioavailability studies.

## Figures and Tables

**Table 1:** *S. transsylvanica* material used for the extraction process and sample labeling according to each harvesting date.

Plant Material	Sample ID	Harvesting Date
***Folium*** **(Leaves)**	ST A1	16 May 2018
	ST A2	23 May 2018
	ST A3	30 May 2018
	ST A4	6 June 2018
	ST A5	13 June 2018
***Flos*** **(Flowers)**	ST B1	16 May 2018
	ST B2	23 May 2018
	ST B3	30 May 2018
	ST B4	6 June 2018
	ST B5	13 June 2018

**Table 2:** Chromatographic and spectral characteristics of identified and tentatively annotated phenolic compounds from the *S. transsylvanica* samples, representing leaf (STA1–5) and flower (STB1–5) extracts. The “+” symbol represents successful identification based on spectral characteristics, while “–” represents a lack of identification.

Peak	Rt (min)	λ_max_ (nm)	[M-H]^−^ (*m*/*z*)	MS^n^ (*m*/*z*)	Tentative Identification	Level ^1^	Metabolite Presence in ST Sample									
							A1	A2	A3	A4	A5	B1	B2	B3	B4	B5
**1**	4.48	281	197	179 *, 135 *	Danshensu (salvianic acid A)	2	+	+	+	+	+	+	+	+	+	+
**2**	5.71	260	417	265(40), 221(28), 211 *, 169(100)	Gallic acid derivative	3	+	+	+	+	+	+	+	+	+	+
**3**	9.16	321	297	179(52), 161 *, 135(100)	Caffeoyl-threonic acid	2	+	+	+	+	+	+	+	+	+	+
**4**	10.52	323	179	135(100)	Caffeic acid	1	+	+	+	+	+	+	+	+	+	+
**5**	11.38	276	571	553(26), 527(99), 179(23)	Yunnaneic acid E	2	+	–	–	–	–	–	–	–	–	–
**6**	14.54	231, 285, 315	555	395 *, 357(100), 313(100), 269 *, 197 *	Salvianolic acid K (isomer 1)	2	–	–	–	–	–	–	+	+	+	–
**7**	16.83	234, 284, 319	555	493(43), 359(25), 313(100), 295 *	Salvianolic acid K (isomer 2)	2	+	+	+	+	+	+	–	–	+	–
**8**	17.68	285, 320	521	359(100), 197(100), 179(6), 161(100)	Rosmarinyl hexoside (isomer 1)	2	+	+	+	+	+	+	–	+	+	+
**9**	18.04	291, 321	521	359(100), 197(100), 179(6), 161(100)	Rosmarinyl hexoside (isomer 2)	2	–	–	–	–	–	+	–	–	+	–
**10**	19.23	266, 347	447	285(100), 199(100), 175(62), 151(11)	Luteolin-*O*-hexoside	2	+	+	+	+	+	+	+	+	+	+
**11**	21.83	233, 288, 322	555	537(4), 493(100), 359(5), 197(75), 179(100), 161(100)	Salvianolic acid K (isomer 3)	2	+	+	+	+	+	+	+	+	+	+
**12**	22.51	328	359	197(100), 179(83), 161(100), 135(14), 123(100)	Rosmarinic acid	1	+	+	+	+	+	+	+	+	+	+
**13**	23.50	223, 268, 334	431	269(100), 227(32)	Apigenin-*O*-hexoside	1	+	+	+	+	+	+	+	+	+	+
**14**	24.61	268, 339	489	447(100), 285(100), 175(70)	Kaempferol-*O*-acetyl-hexoside	2	+	+	+	+	+	–	+	+	+	+
**15**	25.08	337	461	299(100), 284(100), 227(40)	Hispidulin-*O*-hexoside (isomer)	2	–	–	+	+	+	–	+	+	+	+
**16**	26.30	322	489	447(9), 285(100)	Luteolin-*O*-acetyl-hexoside (isomer 1)	2	+	+	+	+	+	–	+	–	–	–
**17**	28.43	276, 316	489	447(9), 285(100)	Luteolin-*O*-acetyl-hexoside (isomer 2)	2	–	–	–	–	+	–	–	–	–	+
**18**	28.63	325	473	431(100), 269(100), 227(20)	Apigenin-*O*-acetyl-hexoside	2	–	–	–	–	–	–	+	+	+	–
**19**	29.30	328	373	197(20), 179(100), 161(88), 135(73)	Methyl rosmarinate	2	+	+	+	+	+	+	+	+	+	+
**20**	31.54	242, 301, 325	537	493(100), 359(100), 197(27), 179(38), 161(100), 135 *	Salvianolic acid I	2	+	+	+	+	+	+	+	+	+	+
							A1	A2	A3	A4	A5	B1	B2	B3	B4	B5

*—Relative abundance < 2. ^1^ Metabolite annotation confidence levels: 1—identification based on analytical standard; 2—putatively annotated based on spectral library matching; 3—putatively characterized based on fragmentation.

**Table 3:** Quantification of the phenolic compounds (mg/g of extract) identified in *S. transsylvanica* leaf (STA1–5) and flower (STB1–5) extracts, expressed as average ± standard deviation (*n* = 2). Different letters within a row indicate significant differences between extracts (two-way ANOVA, Tukey’s test, *p* < 0.05).

	Quantification (mg/g of ST Extract)									
Peak	A1	A2	A3	A4	A5	B1	B2	B3	B4	B5
**1**	1.49 ± 0.02 ^c^	1.18 ± 0.04 ^d^	1.20 ± 0.04 ^d^	1.74 ± 0.01 ^bc^	1.37 ± 0.04 ^d^	1.43 ± 0.02 ^cd^	1.84 ± 0.04 ^b^	3.10 ± 0.10 ^a^	1.87 ± 0.04 ^b^	1.44 ± 0.03 ^c^
**2**	2.70 ± 0.10 ^c^	2.69 ± 0.04 ^c^	2.99 ± 0.06 ^bc^	3.20 ± 0.10 ^b^	3.90 ± 0.02 ^a^	3.16 ± 0.00 ^b^	1.95 ± 0.02 ^e^	2.20 ± 0.03 ^de^	2.49 ± 0.03 ^cd^	1.70 ± 0.01 ^e^
**3**	0.98 ± 0.02 ^c^	1.07 ± 0.02 ^c^	1.75 ± 0.01 ^a^	1.51 ± 0.04 ^ab^	1.58 ± 0.04 ^ab^	0.58 ± 0.02 ^d^	1.45 ± 0.02 ^ab^	1.28 ± 0.00 ^bc^	1.44 ± 0.02 ^ab^	1.07 ± 0.02 ^c^
**4**	0.92 ± 0.01 ^c^	1.17 ± 0.01 ^bc^	1.26 ± 0.02 ^b^	2.04 ± 0.01 ^a^	1.72 ± 0.05 ^a^	1.33 ± 0.01 ^b^	1.14 ± 0.01 ^bc^	0.72 ± 0.01 ^c^	1.32 ± 0.02 ^b^	1.15 ± 0.02 ^bc^
**5**	1.48 ± 0.03	–	–	–	–	–	–	–	–	–
**6**	–	–	–	–	–	–	0.76 ± 0.01 ^b^	0.39 ± 0.01 ^c^	3.47 ± 0.01 ^a^	–
**7**	3.04 ± 0.04 ^a^	2.50 ± 0.10 ^bc^	2.11 ± 0.01 ^d^	2.80 ± 0.02 ^a^	2.45 ± 0.02 ^c^	2.78 ± 0.03 ^ab^	–	–	1.20 ± 0.04 ^e^	–
**8**	4.40 ± 0.10 ^b^	2.19 ± 0.00 ^e^	3.17 ± 0.03 ^d^	5.53 ± 0.01 ^a^	3.90 ± 0.10 ^c^	1.47 ± 0.02 ^f^	–	0.61 ± 0.02 ^h^	0.94 ± 0.01 ^g^	0.62 ± 0.00 ^h^
**9**	–	–	–	–	–	2.70 ± 0.10 ^a^	–	–	0.91 ± 0.02 ^b^	–
**10**	3.27 ± 0.01 ^f^	4.31 ± 0.04 ^d^	6.30 ± 0.10 ^a^	5.28 ± 0.04 ^b^	4.81 ± 0.01 ^c^	2.28 ± 0.04 ^g^	4.90 ± 0.10 ^c^	5.03 ± 0.01 ^bc^	3.96 ± 0.01 ^e^	4.72 ± 0.02 ^c^
**11**	17.60 ± 0.20 ^c^	14.50 ± 0.10 ^d^	17.50 ± 0.10 ^c^	22.40 ± 0.60 ^b^	26.50 ± 0.20 ^a^	7.15 ± 0.02 ^f^	6.90 ± 0.10 ^f^	4.70 ± 0.10 ^h^	7.60 ± 0.10 ^e^	5.96 ± 0.04 ^g^
**12**	39.92 ± 0.16 ^f^	33.30 ± 0.10 ^i^	45.80 ± 0.10 ^d^	68.60 ± 0.30 ^a^	51.60 ± 0.40 ^c^	33.93 ± 0.02 ^h^	51.38 ± 0.03 ^c^	39.39 ± 0.01 ^g^	53.51 ± 0.04 ^b^	40.52 ± 0.01 ^e^
**13**	1.25 ± 0.02 ^g^	1.59 ± 0.03 ^f^	1.79 ± 0.02 ^f^	1.80 ± 0.10 ^f^	1.64 ± 0.01 ^f^	2.20 ± 0.03 ^e^	6.40 ± 0.02 ^c^	6.80 ± 0.10 ^b^	5.50 ± 0.10 ^d^	7.70 ± 0.20 ^a^
**14**	1.42 ± 0.00 ^b^	2.34 ± 0.05 ^a^	2.25 ± 0.04 ^a^	2.03 ± 0.03 ^a^	2.20 ± 0.01 ^a^	–	0.88 ± 0.01 ^c^	0.76 ± 0.01 ^c^	0.74 ± 0.01 ^c^	0.85 ± 0.01 ^c^
**15**	–	–	5.02 ± 0.04 ^a^	2.14 ± 0.03 ^c^	3.98 ± 0.01 ^b^	–	1.48 ± 0.01 ^d^	0.82 ± 0.00 ^e^	0.75 ± 0.00 ^e^	0.87 ± 0.01 ^e^
**16**	1.26 ± 0.00 ^a^	1.27 ± 0.01 ^a^	1.28 ± 0.01 ^a^	1.13 ± 0.01 ^a^	1.12 ± 0.00 ^a^	–	0.76 ± 0.00 ^b^	–	–	–
**17**	–	–	–	–	1.21 ± 0.01 ^a^	–	–	–	–	0.75 ± 0.00 ^b^
**18**	–	–	–	–	–	–	1.13 ± 0.01 ^a^	0.35 ± 0.00 ^b^	0.59 ± 0.00 ^b^	–
**19**	13.43 ± 0.04 ^b^	9.50 ± 0.10 ^f^	16.40 ± 0.30 ^a^	12.40 ± 0.10 ^d^	13.10 ± 0.10 ^c^	8.34 ± 0.04 ^g^	12.43 ± 0.01 ^d^	10.31 ± 0.10 ^e^	12.50 ± 0.20 ^d^	10.30 ± 0.10 ^e^
**20**	3.57 ± 0.01 ^de^	3.74 ± 0.03 ^cd^	5.60 ± 0.20 ^b^	6.88 ± 0.03 ^a^	5.30 ± 0.02 ^b^	3.96 ± 0.05 ^cd^	3.70 ± 0.10 ^d^	3.36 ± 0.04 ^e^	4.21 ± 0.04 ^c^	3.75 ± 0.03 ^d^
**TPA ^1^**	**89.60 ± 0.30 ^d^**	**71.80 ± 0.10 ^f^**	**97.80 ± 0.30 ^c^**	**127.00 ± 0.20 ^a^**	**111.50 ± 0.20 ^b^**	**66.78 ± 0.02 ^g^**	**81.62 ± 0.02 ^e^**	**66.00 ± 0.30 ^h^**	**91.50 ± 0.40 ^d^**	**66.50 ± 0.10 ^i^**
**TF ^2^**	**7.20 ± 0.01 ^h^**	**9.51 ± 0.01 ^g^**	**16.60 ± 0.10 ^a^**	**12.40 ± 0.10 ^e^**	**14.97 ± 0.01 ^c^**	**4.50 ± 0.10 ^i^**	**15.50 ± 0.20 ^b^**	**13.70 ± 0.10 ^d^**	**11.50 ± 0.10 ^f^**	**14.80 ± 0.20 ^c^**
**TPC ^3^**	**96.80 ± 0.30 ^f^**	**81.33 ± 0.10 ^g^**	**114.40 ± 0.40 ^c^**	**139.41 ± 0.04 ^a^**	**126.40 ± 0.20 ^b^**	**71.30 ± 0.10 ^i^**	**97.20 ± 0.30 ^e^**	**79.80 ± 0.20 ^h^**	**103.00 ± 0.40 ^d^**	**81.40 ± 0.10 ^g^**

^1^ TPA: Total Phenolic Acids; ^2^ TF: Total Flavonoids; ^3^ TPC: Total Phenolic Compounds. “–” symbol represents “not detected” or “below the limit of quantification”.

**Table 4:** Results for the determination of in vitro antioxidant potential using three standard assays (ABTS, DPPH, and FRAP). Results were expressed as mg Trolox equivalents/g DW, as the average of triplicate values ± standard deviation (*n* = 3). Different superscript letters represent statistically significant differences between all extracts, considered for each assay (by each column) using one-way ANOVA with post-hoc analysis (Tukey’s test).

Sample ^1^	ABTS (mg TE/g DW)	DPPH (mg TE/g DW)	FRAP (mg TE/g DW)
ST A1	131.00 ± 2.45 ^b^	91.23 ± 0.64 ^ab^	276.28 ± 5.11 ^cd^
ST A2	119.43 ± 1.59 ^c^	85.76 ± 0.95 ^c^	236.67 ± 6.70 ^f^
ST A3	117.07 ± 2.08 ^c^	77.71 ± 0.49 ^d^	266.95 ± 7.96 ^de^
ST A4	139.44 ± 0.58 ^a^	92.24 ± 0.33 ^a^	302.32 ± 5.85 ^abc^
ST A5	138.83 ± 0.67 ^a^	89.73 ± 0.29 ^b^	292.62 ± 6.57 ^abce^
ST B1	85.03 ± 0.85 ^e^	76.46 ± 0.67 ^d^	269.75 ± 4.94 ^de^
ST B2	88.01 ± 0.08 ^de^	76.66 ± 1.05 ^d^	305.14 ± 1.00 ^ab^
ST B3	86.90 ± 0.51 ^de^	77.43 ± 0.42 ^d^	319.88 ± 4.03 ^a^
ST B4	90.36 ± 1.15 ^d^	77.94 ± 0.39 ^d^	285.32 ± 26.48 ^bd^
ST B5	84.29 ± 1.45 ^e^	74.43 ± 0.49 ^e^	283.86 ± 2.94 ^bd^

^1^ ST—*S. transsylvanica* hydroethanolic extract; A—leaf extracts; B—flower extracts.

**Table 5:** Results of enzyme inhibition activities against α-glucosidase and acetylcholinesterase. Results were expressed as IC_50_ values (μg/mL), as the average of triplicate values ± standard deviation (*n* = 3). Different superscript letters represent statistically significant differences between all extracts, considered for each assay (by each column) using one-way ANOVA with post-hoc analysis (Tukey’s test).

Sample ^1^	Enzyme Inhibition Activity (IC_50_, μg/mL)	
	α-Glucosidase	Acetylcholinesterase
ST A1	250.57 ± 6.46 ^g^	829.50 ± 22.65 ^cd^
ST A2	232.90 ± 8.02 ^fg^	760.67 ± 22.93 ^cd^
ST A3	189.50 ± 7.15 ^d^	885.73 ± 57.32 ^d^
ST A4	202.97 ± 4.56 ^de^	792.60 ± 40.26 ^cd^
ST A5	219.23 ± 7.59 ^ef^	732.67 ± 64.15 ^bc^
ST B1	84.48 ± 2.89 ^a^	592.70 ± 33.21 ^ab^
ST B2	143.17 ± 5.09 ^c^	584.30 ± 75.35 ^ab^
ST B3	98.82 ± 5.75 ^ab^	696.43 ± 4.70 ^bc^
ST B4	104.95 ± 5.63 ^ab^	535.50 ± 27.39 ^a^
ST B5	114.47 ± 6.27 ^b^	630.53 ± 26.23 ^ab^
Standard	Acarbose: 122.27	Galantamine: 0.002

^1^ ST—*S. transsylvanica* hydroethanolic extract; A—leaf extracts; B—flower extracts.

## Data Availability

All data are contained within the article. Additional raw data are available from the corresponding author.

## Abbreviations

The following abbreviations are used in this manuscript:

ABTS	2,2′-azino-bis(3-ethylbenzothiazoline-6-sulfonic acid)
AChE	Acetylcholinesterase
DMSO	dimethyl sulfoxide
DPPH	2,2-diphenyl-1-picrylhydrazyl
DW	dry weight
FRAP	ferric-reducing antioxidant power
HPLC	high-performance liquid chromatography
PCA	principal component analysis
RA	rosmarinic acid
TFC	total flavonoid content (AlCl_3_ method)
TF	total flavonoids (cumulative, HPLC)
TPA	total phenolic acids (cumulative, HPLC)
TPC	total phenolic content (Folin–Ciocalteu)
